# Silver Nanoparticles in Dental Biomaterials

**DOI:** 10.1155/2015/485275

**Published:** 2015-01-15

**Authors:** Juliana Mattos Corrêa, Matsuyoshi Mori, Heloísa Lajas Sanches, Adriana Dibo da Cruz, Edgard Poiate, Isis Andréa Venturini Pola Poiate

**Affiliations:** ^1^Department of Operative Dentistry, Fluminense Federal University, 28625-650 Nova Friburgo, RJ, Brazil; ^2^Prosthodontics Department, University of São Paulo, 05508-000 São Paulo, SP, Brazil; ^3^School of Chemistry, Rio de Janeiro Federal University, 21941-909 Rio de Janeiro, RJ, Brazil

## Abstract

Silver has been used in medicine for centuries because of its antimicrobial properties. More recently, silver nanoparticles have been synthesized and incorporated into several biomaterials, since their small size provides great antimicrobial effect, at low filler level. Hence, these nanoparticles have been applied in dentistry, in order to prevent or reduce biofilm formation over dental materials surfaces. This review aims to discuss the current progress in this field, highlighting aspects regarding silver nanoparticles incorporation, such as antimicrobial potential, mechanical properties, cytotoxicity, and long-term effectiveness. We also emphasize the need for more studies to determine the optimal concentration of silver nanoparticle and its release over time.

## 1. Introduction

Silver (Ag) ions or salts are known to have a wide antimicrobial effect [1–4] and they have been used for years [5], in different fields in medicine, including wound dressings [6], catheters [7], and prostheses [8]. Besides being a potent antimicrobial, Ag has many advantages, such as low toxicity and good biocompatibility with human cells [9], long-term antibacterial activity, due to sustained ion release [10], and low bacterial resistance [11].

With the advent of nanotechnology, silver nanoparticles (AgNPs) have been synthesized, and they have shown potent antimicrobial properties [12, 13]. AgNPs have demonstrated unique interactions with bacteria and fungi species [12, 14]; thereafter, they are widely used in medical arena, such as in wound sutures [15], endotracheal tubes [16], surgical instruments [17], and bone prostheses [18].

AgNPs have also been applied in several areas of dentistry, as endodontics [19, 20], dental prostheses [21], implantology [22, 23], and restorative dentistry [24–26]. AgNPs incorporation aims to avoid or at least to decrease the microbial colonization over dental materials, increasing oral health levels and improving life quality.

Because of their small size, AgNPs possess chemical, physical, and biological properties distinctive from those presented by traditional bulk materials [27]. Their smaller particles and large surface area provide potent antibacterial effects at a low filler level, diminishing Ag particle concentration necessary for its efficacy [25, 28–30] and avoiding negative influence on mechanical properties [10, 31, 32].

Other advantage provided by the small size is the possibility of AgNPs to penetrate through cell membranes more readily, resulting in higher antimicrobial activity [33], which is especially important since microorganisms in biofilms are more resistant to antimicrobial agents than planktonic pathogens [34].

The antimicrobial mechanism of AgNPs has been extensively investigated but it remains unclear [35]. It seems that silver ions interact with the peptidoglycan cell wall [34] causing structural changes, increased membrane permeability and, finally, cell death [3]. Further, AgNPs could interact with the exposed sulfhydryl groups in bacterial proteins, avoiding DNA replication [36].

Another important aspect to be studied is the toxicity and biocompatibility of AgNPs. Considering their unique physical and chemical properties, it is likely that these nanoparticles also possess unique toxicity mechanisms [37]. Because of that, a better understanding of AgNPs safety is need, in order to increase their clinical use [38].

Nanotechnology provides a wide range of possibilities to develop new antimicrobial materials [3]. However, there are disadvantages, for example, color change, an important property of dental materials [39]. In this review, we discuss AgNPs incorporation into dental materials, such as composite resin and adhesive systems, acrylic resin, root canal fillings, and implants, highlighting aspects regarding microorganism growth inhibition, cytotoxicity, and physical properties of these modified materials.

## 2. AgNPs Characterization

One important step for the development of AgNPs-containing materials is their characterization. Many studies have analyzed the Ag dispersion [26, 28, 32, 40], through Transmission Electron Microscopy (TEM). This technique allows visualizing how AgNPs spread into the tested material, as well as to verify the particle size.

According to Cheng et al. [28], NAg particles of ~3 nm were clearly visible and well dispersed throughout the polymer matrix. These results were confirmed in a subsequent study [29], in which authors reported NAg sizes ranging from 2 to 5 nm. This very small size allows NAg penetration on dentinal tubules [40], which can represent the possibility of inactivating residual bacteria on dentine. Besides that, it has also been shown that AgNPs were well dispersed in the material with minimal appearance of nanoparticle aggregates [26, 32].

Another important feature to be analyzed is the minimum inhibitory concentration (MIC) of AgNPs. MIC is defined as the lowest concentration of antimicrobial agent at which 90% growth is observed in the medium [46]. Hernández-Sierra et al. [47] used the liquid dilution method to find the MIC of 25 nm-AgNPs against* S. mutans* strains, and the results showed an average MIC of 4.86 ± 2.71 *μ*g/mL, suggesting a higher antimicrobial effect of AgNPs.

In a similar study, Espinosa-Cristóbal et al. [48] tested different AgNPs sizes (8.4 nm, 16.1 nm, and 98 nm) and they reported higher MICs than the abovementioned study: 101.98 ± 72.03 *μ*g/mL, 145.64 ± 104.88 *μ*g/mL, and 320.63 ± 172.83 *μ*g/mL, respectively. This probably occurred because of the methodology, which included sucrose for* S. mutans* growing. Authors also verified that MICs were directly proportional to the particle size; it means, as bigger the size as higher the MIC.

## 3. Forms of Incorporation

AgNPs used in dental materials are incorporated through distinct ways, depending on the type of material. For composite resin and adhesive systems, the most common technique is adding a monomer, usually 2-(tert-butylamino)ethyl methacrylate, in order to improve Ag salt solubility in the resin solution [28, 32, 40]. For dental implants, the process is totally different: Titanium samples are soaked in AgNO_3_ solutions, rinsed with deionized water, dried, and irradiated with UV light from a high-pressure Hg lamp. This process allows producing samples with different Ag concentrations, depending on the AgNO_3_ solution concentration [49].

Another difference is related to the form of AgNP obtainment. In some studies the particles are commercially available, so they are obtained directly from the producer [50–52]. In others, AgNPs are prepared by reduction of AgNO_3_, with NaBO_4_ [22], polyvinylpyrrolidone [21], sodium citrate [44], and gallic acid [48], among others.

## 4. Composite Resin and Adhesive System

Dental caries is still the most common and widespread oral disease, having as the main etiologic agent the acidic attack from cariogenic bacteria, such as* Streptococcus mutans* and* Lactobacillus *spp. [51]. Currently, the most widely dental material used to treat caries lesions is composite resin, especially because of its esthetics and load-bearing properties [53–55]. Hence, many studies have been performed, in order to improve quality and durability of polymeric restorative materials [56–58].

In spite of the notable advances obtained, composite restorations accumulate more biofilm than other restorative materials [59–62]. This is especially important in cases of failures on restoration margin [63]. Actually, although it is wanted, the perfect sealing between the restorative material and the cavity wall often does not occur [32]. It has been shown that there is microleakage on restoration margins, and these gaps can be colonized by oral bacteria, resulting in secondary caries [64], which makes necessary the restoration replacement.

In order to prevent or to diminish biofilm accumulation over composite and in the restorations margins, antimicrobial restorative materials have been developed, especially through the incorporation of AgNPs to composite resins [24–26] and adhesive systems [29, 32, 40–42, 50, 52, 65, 66]. These materials are multiphase substances composed of an organic polymer matrix, filler particles, coupling agent (silane), and the initiator-accelerator of polymerization [67], and AgNPs incorporation is based on the modification in the filler components [61].

A research developed by Cheng et al. [26] reported the effect of AgNPs incorporation, at different concentrations, to a composite resin, in order to investigate its mechanical properties and biofilm formation. In this study, composites were synthesized with AgNPs at 0.028, 0.042, 0.088, and 0.175%. Mechanical properties of composites with AgNPs at 0.028% and 0.042% were similar to those with no AgNPs. Besides that, counts of colony forming units (CFU) for total streptococci and* S. mutans*, using AgNPs at 0.042%, were 75% smaller than the control group without AgNPs. These data suggest that AgNPs incorporation to composite resins enables good mechanical properties and notable antimicrobial potential, even at low concentration.

In order to evaluate the influence of AgNPs incorporation on bond strength to dental substrate, Melo et al. [41] added AgNPs, at 0.1% by mass, to an adhesive system. The results have shown that AgNPs did not compromise the bond strength (*P* > 0.1), at the same time that it decreased metabolic activity on biofilm, compared to control group without AgNPs. In this study it was also observed reduction of CFU for total microorganisms, total streptococci, and mutans streptococci (*P* < 0.05).

Li et al. [32] performed a study incorporating of AgNPs, at 0.05% by mass, to an adhesive system, aiming to assess bacterial inhibition provided by this antimicrobial, in both short and long distance. It has been reported that AgNPs reduced CFU number and acid lactic production on biofilm over and away to the adhesive surface, evidencing that AgNPs-containing adhesives enable long-distance antibacterial potential.

Another important aspect to be assessed is the biocompatibility of AgNPs-containing restorative materials. Accordingly, Zhang et al. [42] have studied the effects of AgNPs incorporation, at 0.05% by mass, to a primer and an adhesive, regarding human gingival fibroblast viability. It has been shown that AgNPs addition did not affect the cytotoxicity of primer and adhesive tested, evidencing the clinical applicability of this antimicrobial.

Based on abovementioned studies, it is possible to say that the antibacterial effects of AgNPs-containing restorative materials might decrease the development of recurrent caries, to increase the longevity of tooth restorations, and to be effective in decreasing the formation of bacterial biofilms on teeth and restorations, without compromising mechanical properties and cytotoxicity of composite resins and adhesive systems.

## 5. Acrylic Resin

Dentures, mostly constituted by poly(methyl methacrylate) (PMMA) acrylic resin [68], have their inner surface considerably rough [69], and this roughness, allied to other factors (e.g., poor hygiene, xerostomy, and HIV infection), contributes to the emergence of denture stomatitis [70, 71]. This pathology, characterized by red focal area, mostly localized in palatal mucosa, is present in 50–70% of complete denture wearers [72, 73], and it is frequently associated with* Candida* species colonization. These fungi colonize denture surfaces forming a biofilm [74], which acts as a key-factor to denture stomatitis development [75].

The treatment of denture stomatitis is based on topical or systemic antifungical drugs, for example, fluoconazole and nystatin [76–78]. However, this infection is often persistent, since antifungical resistance has been reported in* Candida* biofilms [75]. Moreover, it has been observed that* Candida* species present in biofilms are less susceptible to antifungical drugs than planktonic cells [79–81]. Another problem related to denture stomatitis is that many geriatric prosthetic wearers present difficulties on keeping the denture clean, due to their reduced motor dexterity, memory loss, and cognitive impairment [21].

Considering the aforementioned factors, denture stomatitis represents a challenge for dentistry, and methods for its prevention, should be encouraged. Accordingly, AgNPs have been satisfactorily incorporated into polymers used as tissue conditioners and as denture base [82–84]. The action mechanisms of AgNPs-incorporated polymers is still unclear, since some authors attribute the antimicrobial effectiveness to the silver ions release [85, 86] and others to the direct contact between the material and the microorganisms [87].

Acosta-Torres et al. [43] developed a PMMA containing 1 *μ*g/mL of AgNPs and they compared this new compound to unmodified PMMA. It has been observed that PMMA-AgNPs specimens showed significantly less* Candida albicans* adherence compared to PMMA (*P* < 0.05), demonstrating the antifungical potential of AgNPs incorporated to acrylic resin. Besides that, they evaluated the activity of mouse fibroblasts and human lymphocytes, and it has been shown that PMMA-AgNP compound does not present cytotoxity or genotoxicity. These results suggest that the novel acrylic resin incorporated with AgNPs could be developed as a denture base.

In a study performed by Monteiro et al. [44] AgNPs were incorporated in a commercial acrylic resin, in different concentrations (0.05%, 0.5%, and 5% of AgNPs, by mass). The authors evaluated the mechanical properties of the modified resin, as well of the unmodified one (0% of AgNPs). Thereunto, the flexural strength test was performed, and it was observed that all the groups presented very similar flexural resistance values, suggesting that AgNPs incorporation does not affect the mechanical properties of acrylic resin.

When dentures are ill-fitted is recommended recovering his base with tissue conditioners, which are easily degradable with time and occasionally susceptible to microbial colonization [88]. Thus, AgNPs incorporation could also be profitable in this material and not only in dentures base.

Accordingly, Nam [21] has incorporated AgNPs into a commercial tissue conditioner, in the following concentrations: 0.1%, 0.5%, 1.0%, 2.0%, and 3.0%. Their inhibitory effect was evaluated against* Staphylococcus aureus, Streptococcus mutans*, and* Candida albicans* after 24 h and 72 h. The authors have reported that the modified tissue conditioner presented antimicrobial properties even at lower concentrations, that is, 0.1% (for* S. mutans* and* S. aureus*) and 0.5% (for* C. albicans*).

## 6. Endodontic Materials

Several studies have demonstrated that bacteria are the main etiologic agent of pulpal infection and periradicular lesion formation [89–91]. The microbiota of infected root canals is polymicrobial and is dominated by Gram-negative anaerobes [92, 93]. It has been demonstrated that the presence of residual bacteria in root canal is connected with significantly higher rates of treatment failure [94].

Since elimination of bacteria in root canals is the key to treatment success [95], endodontic materials should ideally provide some antimicrobial activity [96, 97], in order to improve the prognosis of endodontically treated teeth [98]. Various materials have been used as root canal fillings, among which gutta-percha is one of the most used [95]. This material has been proved to present slight antibacterial property, provided by the zinc oxide in its components; however, this does not provide to gutta-percha an effective bactericidal potential [98].

Accordingly, Iranian researchers [45] have introduced nanosilver-gutta-percha, as an attempt to improve the antibacterial effect of gutta-percha. The new material, which is standard gutta-percha coated with AgNPs, has demonstrated significant effect against* Enterococcus faecalis*,* Staphylococcus aureus*,* Candida albicans*, and* Escherichia coli*.

Besides that, Shantiaee et al. [99] have tested the biocompatibility of this new material, by comparing the cytotoxicity of nanosilver-coated gutta-percha and normal gutta-percha on mouse fibroblasts. In this study, after 24 hours, nanosilver-coated gutta-percha presented cytotoxicity similar to normal gutta-percha and, after one week, it reached the lowest level of cytotoxicity among the tested materials.

Other important step in the endodontic treatment is the chemomechanical debridement of pulpal tissue and pathogenic bacteria. In this stage, irrigant solutions should be used, for dissolving tissue and disinfecting the root canal system [100]. For this purpose, sodium hypochlorite (NaOCl) has been used for more than 70 years, and it remains as one of the most common solutions [101]. However, if NaOCl passes beyond the apex, it is extremely toxic to the periapical tissues [102].

In this context, Lotfi et al. [20] performed a study comparing the antibacterial effect of NaOCl and AgNP solution against* Enterococcus faecalis*, which is a bacterium often isolated from failed endodontic treatment cases [103]. Authors have observed that there were no significant differences among 5.25% NaOCl and 0.005% AgNPs, suggesting that this solution, in a remarkably lower concentration, possesses the same bactericidal effect as 5.25% NaOCl; hence, it could be used as a new intracanal irrigant.

Another important endodontic material is the mineral trioxide aggregate (MTA), used in many indications such as perforations sealing, external/internal root resorption repair, and apexification [104, 105]. In spite of being a material of wide application, the antimicrobial properties of MTA are controversial, and they seem to be limited [106, 107].

Aiming to improve its antimicrobial potential, Samiei et al. [19] modified MTA by adding AgNPs, at 1% weight. Its effect against oral bacteria and fungi species was assessed. Results have showed that AgNPs-containing MTA possesses higher antimicrobial effect against* Enterococcus faecalis, Candida albicans*, and* Pseudomonas aeruginosa*, compared to unmodified MTA.

Although AgNP is a promising antimicrobial, there are only a few studies employing it in endodontic materials. And considering that endodontic treatment success is highly connected to the bacteria elimination, researches involving AgNPs incorporation to root canal filling materials and intracanal irrigators should be encouraged.

## 7. Titanium Implants

Titanium (Ti) implants, widely used in dentistry, usually present infection around their surface, which remains one of the most important complications in Implantology [49, 108]. Several measures have been proposed to avoid bacterial contamination, such as implant disinfection and aseptic surgical protocols; nevertheless, bacterial invasion often occurs after surgery [109].

In order to prevent biofilm formation over implants surface, antibacterial coatings have been developed; however, most of them present poor long-term antibacterial action and also the possibility of generating resistant strains after prolonged use [110–112]. In this context, AgNPs incorporation to implant surface has been suggested [109, 113], since it would be possible to produce coatings with long-term antibacterial properties by controlling Ag release [23].

In study performed by Zhao et al. [23], AgNPs were incorporated into titania nanotubes (TiO2-NTs) on Ti implants, in a process involving silver nitrate immersion and ultraviolet radiation. The antibacterial effect against* Staphylococcus aureus* was assessed, and results have shown inhibition of planktonic bacteria during the first several days. Moreover, AgNPs-coating Ti implants have presented ability to prevent bacteria adhesion for up to 30 days, which are considered sufficient time to prevent post-infection in early stages.

In a similar study, Flores et al. [22] have evaluated the antibacterial activity of AgNPs against* Pseudomonas aeruginosa*. It has been reported that the number of total cells found on AgNP-modified implants represents only 20% of those attached to unmodified surfaces. This data suggests that the incorporation of AgNPs on Ti implants is an efficient method to protect implant surface against pathogen colonization.

As important as the antibacterial potential is the biocompatibility of these modified implants. Aiming to evaluate this property, Lu et al. [114] have tested Ti implants incorporated with different concentrations of AgNPs (0.5, 1, 1.5, 2 M). For all the tested concentrations, osteoblasts started to adhere on the coatings after 1 day of culture and spread well until 7 days of culture. However, after this, the inhibitory effect of 1 M Ag on cell proliferation became significant, suggesting that AgNP coatings with low amounts of silver were more favorable for osteoblasts growth.

## 8. Future Perspectives

As shown in the previous paragraphs, AgNPs-containing dental materials present good antimicrobial properties (Table 1). However, much is still to be discovered. One of the most important experiments to be performed is to apply the bench results on* in vivo* studies [29, 52], since laboratory conditions do not exactly reproduce oral conditions. Other aspect to be investigated is the long term effectiveness of AgNPs applied on dental materials [32, 51], whereas a long lasting antimicrobial potential of them is desirable.

## 9. Conclusions

In this review, the antimicrobial effect of AgNPs incorporation into dental materials was investigated, such as composite resin, endodontic materials, acrylic resin, and implants. Several studies have shown that silver, in its nanoparticulated form, possesses an inhibitory effect against many bacteria and fungi, including* S. mutans, C. albicans, P. aeruginosa, E. faecalis, *and* S. aureus*, among others, which could decrease the occurrence of secondary caries, fungical infection, fails on endodontic treatment, and dental implant losses. Although AgNP is a promising antimicrobial to be used in dentistry, its application on some areas, as endodontics and implantology, remains scarce; thereafter, we mostly encourage studies on these fields.

AgNP has also been proved to be biocompatible with mammalian cells, suggesting that its application on dental materials does not represent a threat to human health. However, more studies are necessary to determine the optimal concentration of this silver compound, in order to guarantee the antimicrobial effect without increasing its cytotoxicity. Moreover, further studies are needed to investigate the Ag ion release and long-term properties of the new AgNP-containing dental materials. We also encourage researchers to study and elucidate the best ways of silver incorporation as well as the possible negative influence of its addition in dental materials, especially regarding color changes and mechanical properties.

## Figures and Tables

**Table 1:** Antimicrobial activity of AgNPs-containing dental materials.

Material studied	AgNPs concentration	Antimicrobial effectiveness	Reference
Composite resin	0.028 wt%, 0.042 wt%, 0.088 wt%, 0.175 wt%	Good inhibitory activity against *S. mutans*, at 0.042 wt%	[25]
Adhesive system	0.1 wt%	Reduction of CFU for total microorganisms, total streptococci, and *S. mutans *	[41]
Adhesive system	0.05 wt%	Reduction of CFU and acid lactic production for *S. mutans *	[32]
Primer and adhesive	0.05 wt%	Good inhibitory activity against total microorganisms, total streptococci, and *S. mutans *	[42]
Acrylic resin	1 *µ*g/mL	Reduction on *C. albicans *adherence	[43]
Acrylic resin	0.05 vol%, 0.5 vol% and 5 vol%	Good efficacy against *C. albicans*, at 5 vol%	[44]
Tissue conditioner	0.1%, 0.5%, 1.0%, 2.0% and 3.0%.	Antimicrobial properties against *S. mutans* and *S. aureus* at 0.1% and against *C. albicans* at 0.5%	[21]
Intracanal irrigant	0.005%	Bactericidal effect against *E. faecalis *	[20]
Gutta-percha	Not mentioned	Significant effect against *E. faecalis, S. aureus*, *C. albicans* and *E. coli*.	[45]
MTA	1 wt%	High antimicrobial effect against *E. faecalis, C. albicans*,and *P. aeruginosa *	[19]
Titanium implants	0.5 M, 1.0 M, 1.5 M, 2.0 M	Prevention of *S. aureus* adhesion for up to 30 days	[23]
Titanium implants	3.16 × 10^−2^ mg Ag/mL	Reduction of *P. aeruginosa* adhesion	[22]
